# *Robiginitalea rubriflava* sp. nov. and *Robiginitalea insularis* sp. nov., isolated from coastal seawaters of the Yellow Sea

**DOI:** 10.71150/jm.2512009

**Published:** 2026-01-28

**Authors:** Seungyeop Oh, Yeonjung Lim, Meora Rajeev, Jang-Cheon Cho

**Affiliations:** 1 Department of Biological Sciences and Bioengineering, Inha University, Incheon 22212, Republic of Korea; 2 Center for Molecular and Cell Biology, Inha University, Incheon 22212, Republic of Korea; 3 Institute for Specialized Teaching and Research, Inha University, Incheon 22212, Republic of Korea

**Keywords:** *Robiginitalea rubriflava*, *Robiginitalea insularis*, polyphasic taxonomy, marine bacteria, novel species, genome

## Abstract

Two Gram-stain-negative, aerobic, non-motile, rod-shaped bacterial strains, designated IMCC43444^T^ and IMCC44478^T^, were isolated from surface seawater collected off Deokjeok Island and Jangbong Island, respectively, in the Yellow Sea. The two strains shared 100% 16S rRNA gene sequence similarity with each other but exhibited ≤ 96.2% similarity to validly published species of the genus *Robiginitalea*. Complete whole-genome sequences of IMCC43444^T^ and IMCC44478^T^ were 3.21 Mb and 3.30 Mb in size, with DNA G + C contents of 46.5% and 46.4%, respectively. Genome-based relatedness analyses revealed average nucleotide identity (ANI) and digital DNA–DNA hybridization (dDDH) values of 90.7% and 42.9% between the two strains, which are well below the accepted species-level thresholds. Furthermore, ANI (≤ 70.2%) and dDDH (≤ 17.8%) values relative to type strains of *Robiginitalea* species supported the conclusion that strains IMCC43444^T^ and IMCC44478^T^ each represent novel species within the genus. Chemotaxonomic characterization showed that iso-C_15:0_, iso-C_17:0_ 3-OH and iso-C_15:1_ G were the major fatty acids of both strains; menaquinone-6 (MK-6) was the sole isoprenoid quinone; and the major polar lipids comprised phosphatidylethanolamine, glycolipids, aminolipids, phospholipids, and other unidentified lipids. Based on phylogenetic, genomic, and phenotypic evidence, strains IMCC43444^T^ and IMCC44478^T^ are proposed as two novel species, *Robiginitalea rubriflava* sp. nov. and *Robiginitalea insularis* sp. nov., respectively. The type strains are IMCC43444^T^ (= KCTC 102397^T^ = JCM 37893^T^) and IMCC44478^T^ (= KCTC 102398^T^ = JCM 37894^T^).

## Introduction

The genus *Robiginitalea*, belonging to the family *Flavobacteriaceae* within the class *Flavobacteriia*, was first proposed by Cho and Giovannoni (2004) based on seawater isolates from the Sargasso Sea in the Atlantic Ocean, and currently comprises six validly published species: *R. biformata* (the type species; Cho and Giovannoni, 2004), *R. myxolifaciens* (Manh et al., 2008), *R. sediminis* (Zhang et al., 2018), *R. marina* (Xuan et al., 2022), *R. aurantiaca* (Zhou et al., 2023), and *R. aestuariiviva* (Cao et al., 2023). The six currently recognized species of the genus *Robiginitalea* have been recovered from a broad array of marine environments, including open-ocean seawater, coastal sedimentary habitats, sea-cucumber aquaculture ponds, and tidal-flat sediments, reflecting the ecological versatility of the lineage.

The species of the genus *Robiginitalea* share several conserved phenotypic characteristics, as they are Gram-stain-negative, aerobic, and non-motile bacteria that characteristically produce pale red to orange pigments and grow optimally under mesophilic and moderately halophilic conditions typical of coastal marine ecosystems. Chemotaxonomically, the presence of menaquinone-6 (MK-6) as the predominant respiratory quinone, together with phosphatidylethanolamine (PE) as a major identified polar lipid, in the genus *Robiginitalea* reflects a chemotaxonomic profile broadly shared among members of the family *Flavobacteriaceae* (Bernardet et al., 2002). Genomic features of the genus *Robiginitalea* also fall within a relatively narrow range, with genome sizes of 3.21–3.53 Mb and DNA G + C contents of 46.9–55.7 mol%. Notably, these comparatively high G + C values place the genus among the upper range within the family *Flavobacteriaceae*, most members of which typically exhibit substantially lower G + C contents of approximately 30–45 mol% (Gavriilidou et al., 2020; Kim et al., 2022). Morphological diversity within the genus is noteworthy, as species such as *R. biformata*, *R. myxolifaciens*, *R. sediminis*, and *R. aestuariiviva* exhibit pleomorphic transitions between rod-shaped and coccoid cells, whereas *R. marina* and *R. aurantiaca* maintain consistently rod-shaped morphology, suggesting potential ecological or genomic determinants underlying morphological stability.

Coastal waters of the Korean Peninsula are recognized as biologically dynamic systems that harbor a substantial reservoir of uncharacterized microbial lineages, and numerous novel bacterial taxa have indeed been intermittently isolated and described from these environments in recent years (Han et al., 2025; Kim et al., 2025; Lee et al., 2024; Tak et al., 2024; Yang et al., 2024). As part of our ongoing investigation into marine bacterial diversity, we characterized strains IMCC43444^T^ and IMCC44478^T^, which were isolated from coastal surface seawater off two islands in the Yellow Sea during a survey of prokaryotic diversity in island-associated marine habitats. Comprehensive analyses of their physiological, phylogenetic, chemotaxonomic, and genomic characteristics supported the conclusion that each of the two strains represents a novel species within the genus *Robiginitalea*.

## Materials and Methods

### Isolation and culture conditions

Strain IMCC43444^T^ was isolated from coastal surface seawater collected at Deokjeok Island, Incheon, Republic of Korea (37.2211º N, 126.1126º E) in June 2022, and strain IMCC44478^T^ was obtained from Jangbong Island, Incheon (37.5283º N, 126.3469º E) in June 2023. Serial dilutions of the seawater samples were spread onto marine agar 2216 (MA; BD Difco, USA), and colonies obtained after incubation at 25°C for 5 days were purified by three successive transfers. The purified isolates were maintained on MA and preserved as 10% (v/v) glycerol suspensions at −80℃. For comparative phenotypic analyses, the closely related type strains *R. biformata* KCTC 12146^T^ (= HTCC2501^T^) and *R. sediminis* KCTC 52898^T^ (= O458^T^) were obtained from the Korean Collection for Type Cultures (KCTC) and cultivated on MA at 25°C for 5 days.

### Whole-genome sequencing and analysis

Approximately 50 mg (wet weight) of cells of strains IMCC43444^T^ and IMCC44478^T^ harvested from MA plates was resuspended in DNA/RNA Shield (Zymo Research, USA) and shipped to MicrobesNG (UK) for sequencing. Whole-genome sequencing was carried out by MicrobesNG using a hybrid approach that combined Illumina and Oxford Nanopore platforms. Short-read libraries were generated with the Nextera XT DNA Library Preparation Kit (Illumina, USA) and sequenced on an Illumina NextSeq 2000 instrument to produce 250 bp paired-end reads. Long-read libraries were prepared using the SQK-RBK114.96 kit (Oxford Nanopore Technologies, UK) and sequenced on a PromethION system equipped with a FLO-PRO114M flow cell. Hybrid genome assembly was generated with Hybracter v0.11.2 (Bouras et al., 2024). Genome completeness and contamination were assessed using CheckM2 v1.1.0 (Chklovski et al., 2023) with default parameters.

For comparative genomic analyses and assessment of genome relatedness, the genome assemblies of *R. biformata* HTCC2501^T^ (accession no. CP001712), *R. myxolifaciens* DSM21019^T^ (FOYQ00000000), *R. sediminis* O458^T^ (NGNR00000000), *R. marina* 2V75^T^ (JAMXIB000000000), *R. aurantiaca* M39^T^ (JAUDUY000000000), and *R. aestuariiviva* M366^T^ (JARPUQ000000000) were retrieved from the GenBank database. Average nucleotide identity (ANI) values were determined using JSpeciesWS (Richter et al., 2016), and digital DNA–DNA hybridization (dDDH) values were estimated with the Genome-to-Genome Distance Calculator (GGDC 3.0) (Meier-Kolthoff et al., 2013). Genome-based phylogenetic relationships were inferred using GTDB-Tk v2.4.0 (Chaumeil et al., 2022) with the bacterial bac120 marker gene set from the Genome Taxonomy Database (GTDB) release R226. The resulting concatenated alignment of 120 single-copy marker genes was analyzed under the maximum-likelihood framework using RAxML v8.2.12 (Stamatakis, 2014). Functional genome annotation was performed with Prokka v1.14.6 (Seemann, 2014), after which predicted protein sequences were assigned to KEGG orthologs using BlastKOALA (Kanehisa et al., 2016), and metabolic pathway profiles were refined with kegg-pathway-completeness tool v1.3.0 (https://github.com/EBI-Metagenomics/kegg-pathways-completeness-tool).

### 16S rRNA gene-based phylogenetic analysis

Genomic DNA from strains IMCC43444^T^ and IMCC44478^T^ was extracted by suspending purified colonies in Tris-EDTA buffer, followed by multiple freeze–thaw cycles and mechanical disruption with glass beads. The resulting lysates were used directly as templates for PCR amplification of the 16S rRNA gene using the universal bacterial primers 27F and 1492R. PCR products were sequenced by Macrogen, Inc. (Korea) using the Sanger method with the sequencing primers 518F, 519R, 800R, and 926F. Since the genome-derived 16S rRNA gene sequences were identical to the PCR-amplified sequences, subsequent analyses were conducted using the genome-derived sequences (see ‘Results and Discussion’).

The 16S rRNA gene sequences were compared against the GenBank database using BLASTn, and pairwise similarity values were calculated with the EzBioCloud server (Chalita et al., 2024). For phylogenetic analyses, the 16S rRNA gene sequences of the two strains and those of related taxa were aligned with the SINA aligner (SILVA Incremental Aligner; v1.2.12) (Pruesse et al., 2012) and imported into the ARB software package (Ludwig et al., 2004). Phylogenetic trees were reconstructed in MEGA X (Kumar et al., 2018) using three algorithms: maximum-likelihood (ML) (Felsenstein, 1981) with the Tamura–Nei substitution model (Tamura and Nei, 1993), neighbor-joining (NJ) (Saitou and Nei, 1987), and minimum-evolution (ME) (Fitch, 1971), using evolutionary distances calculated with the Jukes–Cantor model (Jukes and Cantor, 1969). The robustness of tree topologies was evaluated by bootstrap analyses with 1,000 replicates (Felsenstein, 1985).

### Physiological and biochemical characterization

Cell morphology was examined by transmission electron microscopy (CM200, Philips, Netherlands) using a negative-staining procedure. Cells were suspended in 0.2 M cacodylate buffer, stained with 1% (w/v) uranyl acetate (Electron Microscopy Sciences, USA), and mounted onto Formvar-coated copper grids (FCF300-CU; Ted Pella, USA). The Gram reaction was assessed using the KOH-based non-staining method (Powers, 1995). Catalase activity was tested by applying 3% (v/v) hydrogen peroxide, whereas oxidase activity was determined using 1% (w/v) Kovac’s reagent (bioMérieux, France). Carotenoid pigments were extracted with acetone, and absorption spectra were scanned over the range of 350–600 nm using a UV–Vis spectrophotometer (UV-2600, Shimadzu, Japan). Flexirubin-type pigments were examined using the KOH method (Fautz and Reichenbach, 1980) by applying a 20% (w/v) KOH solution to fresh colonies and resulting color changes were recorded. Motility was evaluated by inoculating cells into soft marine agar (0.5%, BD Difco, USA) and monitoring the outward dispersion of growth. Growth at various temperatures was examined on MA plates at 4°C and from 10 to 50°C at 5°C intervals. Salt requirements were determined on NaCl-free MA supplemented with NaCl to final concentrations of 0–4.0% (0.5% increments), 5%, 7.5%, 10%, 15%, and 20% (w/v). The pH range and optimum were assessed in marine broth (MB, BD Difco, USA) adjusted to pH 3.5–10.5 at 0.5-unit intervals using citrate, MES, MOPS, HEPES, Tris, and CHES buffers (all from Sigma-Aldrich, USA). Growth under these conditions was monitored by measuring OD_600_ with a UV-Vis spectrophotometer (UV2600; Shimadzu, Japan). Anaerobic growth was evaluated using the GasPak^TM^ EZ Anaerobe Pouch System with Indicator (BD Diagnostics, USA). Hydrolytic activities were examined on MA containing starch (1%, w/v), carboxymethyl cellulose (1%, w/v), chitin (1%, w/v), casein (3% skim milk, w/v), Tween 20 (1%, v/v), and Tween 80 (1%, v/v) (all from Sigma-Aldrich, USA). For casein-containing plates, skim milk solution and marine agar were autoclaved separately and aseptically mixed prior to plate preparation. DNA degradation was assessed using DNase Test Agar (BD Diagnostics, USA), and hydrogen sulfide (H2S) production was evaluated on Triple Sugar Iron (TSI) Agar (BD Difco, USA). Additional biochemical characteristics were determined using API 20NE and API ZYM kits (bioMérieux, France) and the GEN III MicroPlate system (Biolog, USA), with salinity adjusted to 2% NaCl.

### Chemotaxonomic characterization

Fatty acid methyl ester (FAME) analysis was conducted using biomass from strains IMCC43444^T^, IMCC44478^T^, *R. biformata* HTCC2501^T^, and *R. sediminis* O458^T^ grown on MA plates at 25°C for 5 days. Cellular fatty acids were converted to methyl esters and analyzed on an Agilent 7890 GC (Agilent Technologies, USA) using the Sherlock MIS (MIDI, USA) v6.1 with the TSBA6 database (Sasser, 1990). Polar lipids were extracted following the method of Minnikin et al. (1984) and separated by two-dimensional thin-layer chromatography (TLC) on silica gel 60 F254 plates (Merck, Germany). TLC plates were developed using chloroform/methanol/water (60:30:4, v/v) in the first dimension and chloroform/acetic acid/methanol/water (40:7.5:6:1.8, v/v) in the second dimension. Total polar lipids were visualized with molybdophosphoric acid, while specific lipid classes were detected using ninhydrin (aminolipids), molybdenum blue (phospholipids), α-naphthol (glycolipids), and Dragendorff’s reagent (phosphatidylcholine). Respiratory isoprenoid quinones were co-extracted with polar lipids and analyzed by reverse-phase high-performance TLC (HPTLC) on RP-18 F254 plates (Merck, Germany) according to Collins and Jones (1981). Quinone types were identified by comparing their chromatographic mobilities with those of type strain of the type species, *R. biformata* HTCC2501^T^.

### Nucleotide sequence accession numbers

The 16S rRNA gene sequences of strains IMCC43444^T^ and IMCC44478^T^ have been deposited in the GenBank/EMBL/DDBJ databases under accession numbers PX239447 and PX239448, respectively. The complete genome sequences of IMCC43444^T^ and IMCC44478^T^ are available under accession numbers CP199309 and CP199296, respectively.

## Results and Discussion

### 16S rRNA gene-based phylogeny

The PCR-amplified 16S rRNA gene sequences of strains IMCC43444^T^ (1,453 bp) and IMCC44478^T^ (1,442 bp) were completely identical to their corresponding genome-derived sequences (1,524 and 1,525 bp, respectively). Both strains contained two copies of the 16S rRNA gene, which were identical within each genome, and thus subsequent 16S rRNA gene analyses were carried out using the genome-derived sequences. Pairwise sequence analysis revealed that strains IMCC43444^T^ and IMCC44478^T^ shared 100% 16S rRNA gene sequence identity with each other. Because this value clearly exceeds the commonly accepted 98.7% species-level threshold (Riesco and Trujillo, 2024), additional genome-based analyses were necessary to determine whether the two strains represent a single species or two distinct species.

**Fig. 1.**
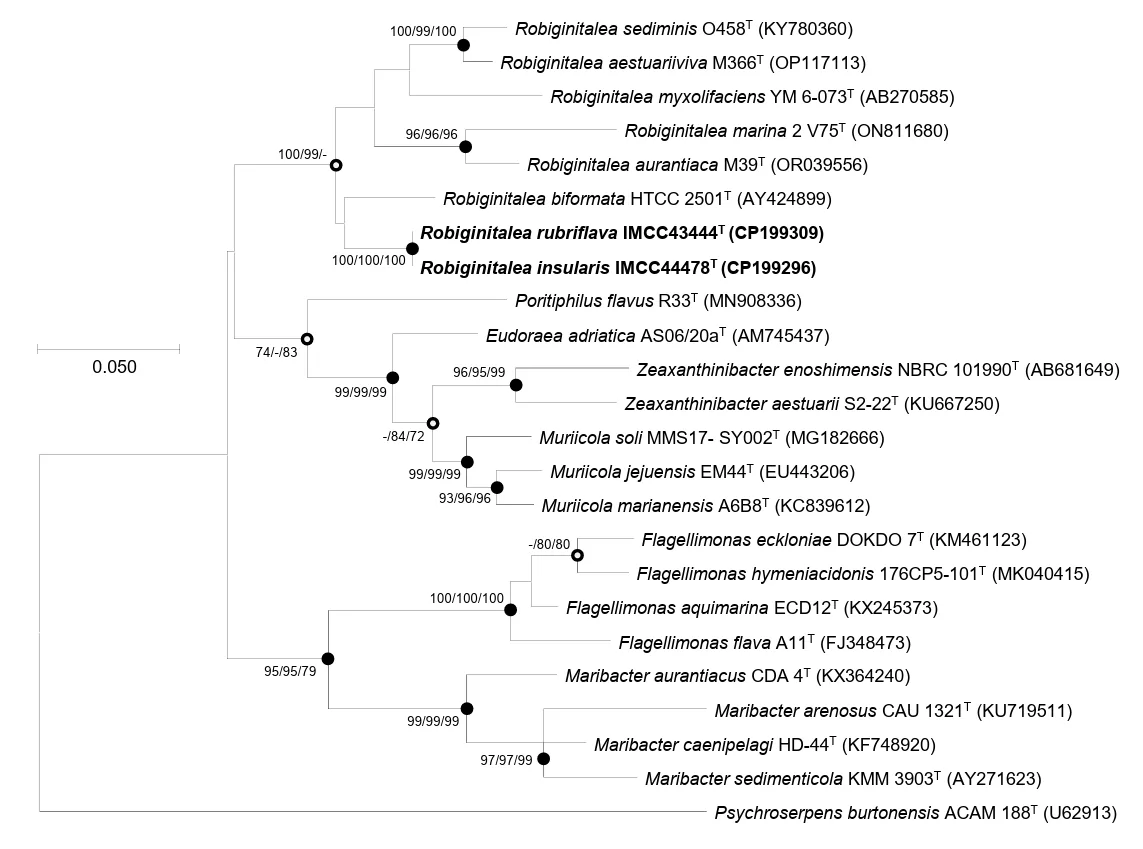
Maximum-likelihood phylogenetic tree based on 16S rRNA gene sequences showing the phylogenetic placement of strains IMCC43444^T^ and IMCC44478^T^. Bootstrap support values (≥ 70%) from the maximum-likelihood, neighbor-joining, and minimum-evolution methods (in that order) are shown at the corresponding nodes. Closed circles indicate nodes recovered by all three tree-building methods, whereas open circles denote nodes supported by two of the three methods. The 16S rRNA gene sequences of strains IMCC43444^T^ and IMCC44478^T^ used for tree reconstruction were derived from the complete genome sequences. Scale bar, nucleotide substitutions per site.

Comparative 16S rRNA gene sequence analyses and their phylogenetic relationships suggested that strains IMCC43444^T^ and IMCC44478^T^ are affiliated with the genus *Robiginitalea*. The two strains exhibited highest similarity to *R. biformata* HTCC2501^T^ (96.2%), followed by *R. sediminis* O458^T^ (95.4%), *R. aurantiaca* M39^T^ (95.2%), *R. aestuariiviva* M366^T^ (95.1%), *R. myxolifaciens* DSM 21019^T^ (94.1%), and *R. marina* 2V75^T^ (94.1%). These similarity values, all below the 98.7% cutoff, clearly indicated that the strains represent a lineage distinct from previously characterized members of the genus *Robiginitalea*. Phylogenetic analysis of the 16S rRNA gene sequences placed strains IMCC43444^T^ and IMCC44478^T^ within the genus *Robiginitalea*, where they formed a distinct and strongly supported clade (Fig. 1). The two strains clustered together on a distinct branch as a sister group to *R. biformata* HTCC2501^T^, consistent with their highest pairwise similarity to this species (96.2%), and were clearly separated from the six validly published *Robiginitalea* species, supporting their placement as a coherent lineage within the genus.

### Genome characteristics and phylogenomic analysis

The complete genomes of strains IMCC43444^T^ and IMCC44478^T^ were each assembled into a single circular chromosome of 3,209,214 bp and 3,300,668 bp, with G + C contents of 46.5% and 46.4%, respectively. Genome quality assessed using CheckM2 indicated completeness and contamination values of 100% and 0.2% for IMCC43444^T^ and 99.9% and 0.1% for IMCC44478^T^, confirming high-quality assemblies. Genome annotation identified 2,887 and 2,968 protein-coding sequences, 39 and 40 tRNA genes, and six rRNA genes in IMCC43444^T^ and IMCC44478^T^, respectively. The two strains notably possess two copies of the *rrn* operon, whereas all previously described *Robiginitalea* species contain only a single copy (Table S1). A summary of the general genomic features of the two strains and the six type strains of validly published *Robiginitalea* species is presented in Table S1.

**Fig. 2.**
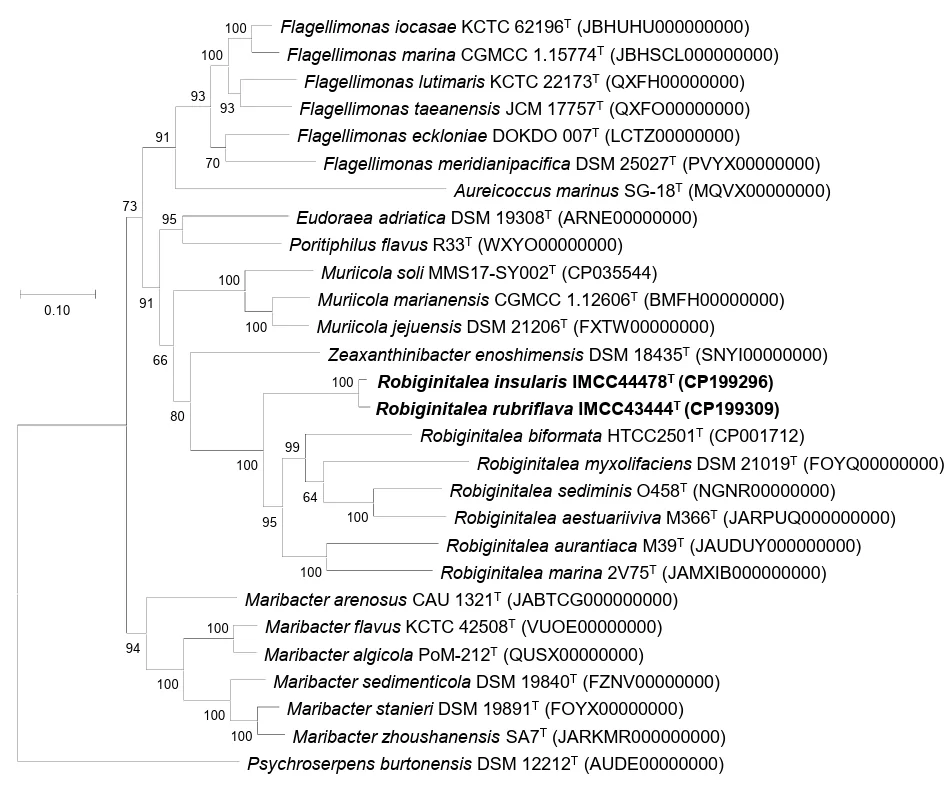
Maximum-likelihood phylogenomic tree showing the relationships between strains IMCC43444^T^ and IMCC44478^T^ and their related type strains. The tree was reconstructed using GTDB-Tk (bac120 marker set) and RAxML based on the concatenated amino acid sequences of 120 conserved single-copy marker genes. Bootstrap support values (≥ 70%) are shown at the nodes. Scale bar, substitutions per amino acid position.

Overall genome relatedness among strains IMCC43444^T^, IMCC44478^T^, and the validly published *Robiginitalea* species was assessed using ANI and dDDH values. The ANI and dDDH values between IMCC43444^T^ and IMCC44478^T^ were 90.7% and 42.9%, respectively, both below the accepted species-level thresholds of 95–96% ANI and 70% dDDH (Riesco and Trujillo, 2024). These results indicate that the two strains represent distinct novel species of the genus *Robiginitalea*. Pairwise comparisons with six validly published *Robiginitalea* species yielded ANI values of 69.1–70.2% and dDDH values of 16.2–17.8% for both strains, further supporting their delineation as two separate species. Genome-based phylogenetic reconstruction consistently placed IMCC43444^T^ and IMCC44478^T^ in a distinct, well-supported clade within *Robiginitalea*, confirming their affiliation with the genus (Fig. 2).

The high-quality genomes of the two strains were subjected to functional annotation. The completeness of major metabolic pathways in IMCC43444^T^, IMCC44478^T^, and six *Robiginitalea* species is summarized in Fig. S1. All genomes encoded the complete set of central carbon metabolic pathways, including glycolysis (Embden–Meyerhof–Parnas pathway), the tricarboxylic acid cycle, gluconeogenesis, and the pentose phosphate pathway, consistent with a heterotrophic lifestyle. Genes associated with a complete oxidative phosphorylation system were also identified, supporting their capacity for aerobic respiration. Notably, IMCC43444^T^ and IMCC44478^T^, as well as other *Robiginitalea* genomes, harbor genes encoding a high-affinity cbb3-type cytochrome *c* oxidase in addition to the canonical prokaryotic cytochrome *c* oxidase, suggesting potential adaptation to low-oxygen or microaerophilic conditions (Zhang et al., 2023). With respect to nitrogen and sulfur metabolism, none of the genomes examined encoded pathways for sulfur oxidation, sulfur reduction, nitrification, or canonical denitrification, indicating that members of the genus are aerobic chemoorganoheterotrophs. However, the gene encoding nitrous oxide reductase (*nos*Z; K00376) was consistently detected in five *Robiginitalea* species but was absent from IMCC43444^T^ and IMCC44478^T^. These findings suggest that some members of the genus may function as partial denitrifiers capable of reducing nitrous oxide to dinitrogen despite lacking upstream denitrification steps (Pold et al., 2025). Overall, the functional profiles of the two strains resemble those of other *Robiginitalea* species, indicating that they are strictly aerobic chemoorganoheterotrophs. Although strains IMCC43444^T^ and IMCC44478^T^ were isolated from geographically proximate islands, they exhibit notable genomic and phenotypic divergence, suggesting fine-scale micro-niche partitioning within spatially heterogeneous marine habitats.

### Phenotypic characteristics

Transmission electron microscopy revealed that cells of strains IMCC43444^T^ and IMCC44478^T^ were rod-shaped, measuring approximately 0.3–0.7 × 1.3–3.8 µm and 0.5–0.6 × 1.9–2.2 µm, respectively (Fig. S2). No flagella were observed, consistent with the absence of flagellar biosynthesis genes in their genomes. The physiological and biochemical characteristics of IMCC43444^T^ and IMCC44478^T^, along with those of two closely related *Robiginitalea* species including the type species of the genus, are summarized in Table 1 and in the species protologues. The two strains exhibited generally similar physiological profiles, including comparable growth ranges, positive oxidase and catalase activities, and the ability to degrade high-molecular-weight substrates, but differed in a few enzyme activities and carbon source oxidation patterns (Table 1). In addition, strains IMCC43444^T^ and IMCC44478^T^ could be differentiated from the two related *Robiginitalea* species based on differences in growth characteristics, casein degradation, selected enzymatic activities such as gelatinase, and carbon source oxidation profiles.

The fatty acid compositions of IMCC43444^T^, IMCC44478^T^, and two related type strains of the genus *Robiginitalea* are summarized in Table 2. All four strains exhibited similar fatty acid profiles, characterized by the predominance of iso-C_15:0_, iso-C_17:0_ 3-OH, and iso-C_15:1_ G, consistent with their affiliation within the same genus. The two strains shared several major fatty acids present at abundances greater than 10%, including iso-C_15:0_ (29.3% and 27.2% in IMCC43444^T^ and IMCC44478^T^, respectively), iso-C_17:0_ 3-OH (13.0% and 17.3%), and iso-C_15:1_ G (15.1% and 20.5%). Despite these similarities, notable quantitative differences were observed, particularly in anteiso-C_15:0_, which accounted for 14.6% in IMCC43444^T^ but only 6.7% in IMCC44478^T^. These differences, together with variations in selected unsaturated fatty acids, provide phenotypic markers that distinguish the two strains from each other as well as from previously described *Robiginitalea* species.

Both strains contained menaquinone-6 (MK-6) as the sole respiratory quinone, consistent with members of the genus *Robiginitalea*. The major polar lipids of IMCC43444^T^ consisted of phosphatidylethanolamine (PE), two unidentified glycolipids (GL), three unidentified aminolipids (AL), three unidentified phospholipids (PL), and five unidentified lipids (Fig. S3). A similar polar lipid profile was observed for IMCC44478^T^, which contained PE, two GL, three AL, two PL, and two unidentified lipids. The occurrence of PE, GL, and AL as diagnostic lipid classes is consistent with polar lipid patterns previously reported for members of the genus *Robiginitalea*. Taken together, the fatty acid compositions, respiratory quinone type, and polar lipid profiles of strains IMCC43444^T^ and IMCC44478^T^ provide congruent chemotaxonomic evidence supporting their assignment to the genus *Robiginitalea*.

### Taxonomic conclusion

Based on 16S rRNA gene phylogeny, overall genome relatedness indices, and comprehensive physiological and chemotaxonomic characteristics, strains IMCC43444^T^ and IMCC44478^T^ are unambiguously assigned to the genus *Robiginitalea*. The two strains exhibited ANI and dDDH values that were well below the accepted species-level thresholds and showed low genomic similarity to all validly published *Robiginitalea* species. These genomic distinctions, together with clear differences in physiological properties, fatty acid composition, and polar lipid patterns, provide consistent evidence that IMCC43444^T^ and IMCC44478^T^ represent two independent and previously undescribed species within the genus *Robiginitalea*. Accordingly, the names *Robiginitalea rubriflava* sp. nov. and *Robiginitalea insularis* sp. nov. are proposed for IMCC43444^T^ and IMCC44478^T^, respectively.

### Description of *Robiginitalea rubriflava* sp. nov.

*Robiginitalea rubriflava* (ru.bri.fla’va. L. adj. *ruber*, red; L. adj. *flavus*, yellow; N.L. fem. adj. *rubriflava*, reddish-yellow, referring to the colony pigmentation).

Cells are Gram-stain-negative, strictly aerobic, and non-motile rods, measuring 0.3–0.7 µm in width and 1.3–3.8 µm in length. Colonies on marine agar are reddish-yellow, circular, and convex. Carotenoid pigments are present. Flexirubin-type pigments are not detected. Growth occurs at 15–35℃ (optimum, 30℃), pH 6.5–7.5 (optimum, pH 7.0), and in the presence of 0.5–5.0% (w/v) NaCl (optimum, 3.0%). Oxidase and catalase activities are positive. Hydrolyzes Tween 20 and Tween 80, but does not hydrolyze casein, colloidal chitin, DNA, starch, or CM-cellulose. In API 20NE tests, esculin hydrolysis, gelatinase, and β-galactosidase are positive, but nitrate reduction, indole production, glucose fermentation, arginine dihydrolase, and urease activities are negative. In API ZYM tests, alkaline phosphatase, esterase (C4), esterase lipase (C8), lipase (C14), leucine arylamidase, valine arylamidase, cystine arylamidase, trypsin, α-chymotrypsin, acid phosphatase, naphthol-AS-BI-phosphohydrolase, α-galactosidase, α-glucosidase, and N-acetyl-β-glucosaminidase activities are positive, whereas β-galactosidase, β-glucuronidase, β-glucosidase, α-mannosidase, and α-fucosidase activities are negative. In the GEN III MicroPlate (Biolog), the following carbon sources are oxidized: D-trehalose, gentiobiose, D-turanose, α-D-glucose, glucuronamide, α-ketoglutaric acid, L-malic acid, bromo-succinic acid, and acetoacetic acid. The predominant fatty acids are iso-C_15:0_, anteiso-C_15:0_, iso-C_17:0_ 3-OH, and iso-C_15:1_ G. The major respiratory quinone is menaquinone-6 (MK-6). The polar lipid profile comprises phosphatidylethanolamine, two unidentified glycolipids, three unidentified aminolipids, three unidentified phospholipids, and five unidentified lipids.

The type strain is IMCC43444^T^ (= KCTC 102397^T^ = JCM 37893^T^ = HNIBRBA19633^T^), isolated from surface seawater off Deokjeok Island, Republic of Korea. The genome size of the type strain is 3,209,214 bp, with a DNA G + C content of 46.5%. The GenBank accession numbers for the 16S rRNA gene sequence and genome sequence are PX239447 and CP199309, respectively.

### Description of *Robiginitalea insularis* sp. nov.

*Robiginitalea insularis* (in.su.la’ris. L. fem. adj. *insularis*, island-dwelling, referring to the isolation of the type strain from coastal waters surrounding Korean islands.).

Cells are Gram-stain-negative, strictly aerobic, and non-motile rods, measuring 0.5–0.6 µm in width and 1.9–2.2 µm in length. Colonies grown on marine agar are reddish-yellow, circular, and convex. Carotenoid pigments are present. Flexirubin-type pigments are not detected. Growth occurs at 15–40℃ (optimum, 35℃), pH 6.5–7.5 (optimum, pH 7.0), and in the presence of 0.5–5.0% (w/v) NaCl (optimum, 2.5%). Oxidase and catalase activities are positive. Hydrolyzes Tween 20 and Tween 80, but does not hydrolyze casein, colloidal chitin, DNA, starch, or CM-cellulose. In API 20NE tests, esculin hydrolysis, gelatinase, and β-galactosidase are positive, but nitrate reduction, indole production, glucose fermentation, arginine dihydrolase, and urease activities are negative. In API ZYM tests, alkaline phosphatase, esterase (C4), esterase lipase (C8), lipase (C14), leucine arylamidase, valine arylamidase, cystine arylamidase, trypsin, α-chymotrypsin, acid phosphatase, naphthol-AS-BI-phosphohydrolase, α-glucosidase, and N-acetyl-β-glucosaminidase activities are positive, whereas α-galactosidase, β-galactosidase, β-glucuronidase, β-glucosidase, α-mannosidase, and α-fucosidase activities are negative. In the GEN III MicroPlate (Biolog), the following carbon sources are oxidized: dextrin, D-trehalose, gentiobiose, D-turanose, α-D-glucose, D-fructose-6-phosphate, glucuronamide, L-malic acid, bromo-succinic acid, and acetoacetic acid. The predominant fatty acids are iso-C_15:0_, iso-C_17:0_ 3-OH, and iso-C_15:1_ G. The major respiratory quinone is menaquinone-6 (MK-6). The polar lipid profile comprises phosphatidylethanolamine, two unidentified glycolipids, three unidentified aminolipids, two unidentified phospholipids, and two unidentified lipids.

The type strain is IMCC44478^T^ (= KCTC 102398^T^ = JCM 37894^T^ = HNIBRBA19634^T^), isolated from coastal seawater off Jangbong Island, Republic of Korea. The genome size of the type strain is 3,300,668 bp, with a DNA G + C content of 46.4%. The GenBank accession numbers for the 16S rRNA gene sequence and genome sequence are PX239448 and CP199296, respectively.

## Figures and Tables

**Table 1.** Differential phenotypic characteristics of strains IMCC43444^T^ and IMCC44478^T^, and closely related type strains of the genus *Robiginitalea* Strains: 1, IMCC43444^T^; 2, IMCC44478^T^; 3, *R. biformata* KCTC 12146^T^; 4, *R. sediminis* KCTC 52898^T^. Data were obtained from this study. All strains positive for degradation of Tween 20 and Tween 80, and enzyme activities for catalase, oxidase, esculin hydrolysis (*β*-glucosidase), PNPG (*β*-galactosidase), alkaline phosphatase, esterase (C4), esterase lipase (C8), lipase (C14), leucine arylamidase, valine arylamidase, cystine arylamidase, trypsin, α-chymotrypsin, acid phosphatase, and naphthol-AS-BI-phosphohydrolase. Detailed carbon source oxidation pattern is summarized in Table S2. +, Positive; ‒, negative.

Characteristics	1	2	3	4
**Colony color**	reddish-yellow	reddish-yellow	rust-colored	orange
**Growth at**				
Temperature range (optimum, ºC)	15–35 (30)	15–40 (35)	10–45 (30)	15–45 (35)
pH range (optimum)	6.5–7.5 (7.0)	6.5–7.5 (7.0)	6.0–9.0 (8.0)	6.5–8.5 (7.5)
NaCl range (optimum, %)	0.5–5.0 (3.0)	0.5–5.0 (2.5)	0.25–10.0 (2.5)	1.0–6.0 (2.0)
**Hydrolysis of:**				
Casein	–	–	–	+
**API 20NE**				
Gelatinase	+	+	–	+
**API ZYM**				
*α*-Galactosidase	+	–	+	+
*β*-Galactosidase, *α*-mannosidase	–	–	+	–
*β*-Glucuronidase	–	–	–	+
*β*-Glucosidase	–	–	+	+
**GEN III**				
D-Maltose, D-cellobiose, sucrose, *β*-methyl-D-glucoside	–	–	–	+
L-Galactonic acid lactone,	–	–	+	–
L-histidine,				
D-Glucuronic acid	–	–	+	+
Dextrin, D-fructose-6-phosphate	–	+	+	+
D-Trehalose	+	+	–	+
*α*-Ketoglutaric acid	+	–	+	+

**Table 2.** Cellular fatty acid compositions of strains IMCC43444^T^ and IMCC44478^T^ and the closely related type strains of the genus *Robiginitalea* Strains: 1, IMCC43444^T^; 2, IMCC44478^T^; 3, *R. biformata* KCTC 12146^T^; 4, *R. sediminis* KCTC 52898^T^. Data were obtained from this study. All strains were cultured under identical conditions. Fatty acids comprising < 0.5% of the total fatty acid content in all species were omitted. ‒, Not detected; Tr, traces (< 0.5%). Major fatty acids (> 10%) are shown in bold.

Fatty acid (%)	1	2	3	4
**Saturated**				
C_16:0_	1.1	1.8	1.8	1.4
**Branched**				
iso-C_13:0_	0.6	0.6	0.9	1.3
iso-C_14:0_	1.2	0.7	–	1.4
iso-C_15:0_	**29.3**	**27.2**	**22.1**	**23.2**
iso-C_16:0_	3.8	3.6	0.6	1.6
iso-C_17:0_	Tr	0.9	Tr	Tr
anteiso-C_15:0_	**14.6**	6.7	3.9	3.6
**Hydroxy**				
iso-C_15:0_ 3-OH	4.2	4.2	6.4	5.5
iso-C_16:0_ 3-OH	3.4	3.7	2.2	3.4
iso-C_17:0_ 3-OH	**13.0**	**17.3**	**25.4**	**21.0**
C_15:0_ 2-OH	1.7	0.8	0.7	1.0
C_17:0_ 2-OH	2.5	1.8	1.5	1.0
C_15:0_ 3-OH	Tr	Tr	0.7	0.9
C_17:0_ 3-OH	Tr	Tr	0.5	0.7
C_16:0_ 3-OH	1.3	1.2	0.7	1.3
**Unsaturated**				
iso-C_15:1_ G	**15.1**	**20.5**	**21.2**	**23.5**
anteiso-C_15:1_ A	1.7	1.6	0.7	1.1
C_16:1_ iso G	0.5	0.6	–	Tr
C_13:1_ at position 12–13	Tr	Tr	Tr	0.5
C_15:1_ ω6c	Tr	–	0.6	–
C_17:1_ ω6c	Tr	Tr	0.9	–
**Summed feature**				
Summed feature 3^*^	1.8	2.5	6.2	5.8
Summed feature 9^*^	1.1	1.6	1.0	–

* Summed feature 3 consisted of C_16:1_ ω7c and/or C_16:1_ ω6c and summed feature 9 consisted of iso-C_17:1_ ω9c and/or C_16:0_10-methyl.

## Supplementary Information

Table S1. Comparative genomic features of IMCC43444^T^, IMCC44478^T^ and six validly published *Robiginitalea* species

Table S2. Carbon source oxidation patterns (GENⅢ MicroPlate) of IMCC43444^T^, IMCC44478^T^, *Robiginitalea biformata* KCTC 12146^T^, and *Robiginitalea sediminis* KCTC 52898^T^

Fig. S1. Genomic potential of strains IMCC43444^T^ and IMCC44478^T^. Heatmaps depict the completeness of key metabolic pathways involved in central carbon metabolism, nitrogen metabolism, sulfur metabolism, and carbon fixation across these strains and the related type strains of the genus *Robiginitalea*. Numbers in parentheses indicate the corresponding KEGG module accession identifiers.

Fig. S2. Transmission electron micrographs of cells of strains IMCC43444^T^ (A) and IMCC44478^T^ (B) after negative staining with 1.0% (w/v) uranyl acetate. Cells were grown on marine agar at 25°C for 5 days. Bar, 0.5 μm.

Fig. S3. Two-dimensional thin layer chromatograph of polar lipids of strains IMCC43444^T^ (A) and IMCC44478^T^ (B). PE, phosphatidylethanolamine; GL, unidentified glycolipid; AL, unidentified aminolipid; PL, unidentified phospholipid; L, unidentified lipid.
