# Supplementary material for: Robiginitalea rubriflava sp. nov. and Robiginitalea insularis sp. nov., isolated from coastal seawaters of the Yellow Sea
Source: J Microbiol. 2026 Jan 28;64(2):e2512009. doi: 10.71150/jm.2512009 (PMC13577070; doi:10.71150/jm.2512009)

Fig. S1. Genomic potential of strains IMCC43444^T and IMCC44478^T. Heatmaps depict the completeness of key metabolic pathways involved in central carbon metabolism, nitrogen metabolism, sulfur metabolism, and carbon fixation across these strains and the related type strains of the genus *Robiginitalea*. Numbers in parentheses indicate the corresponding KEGG module accession identifiers.

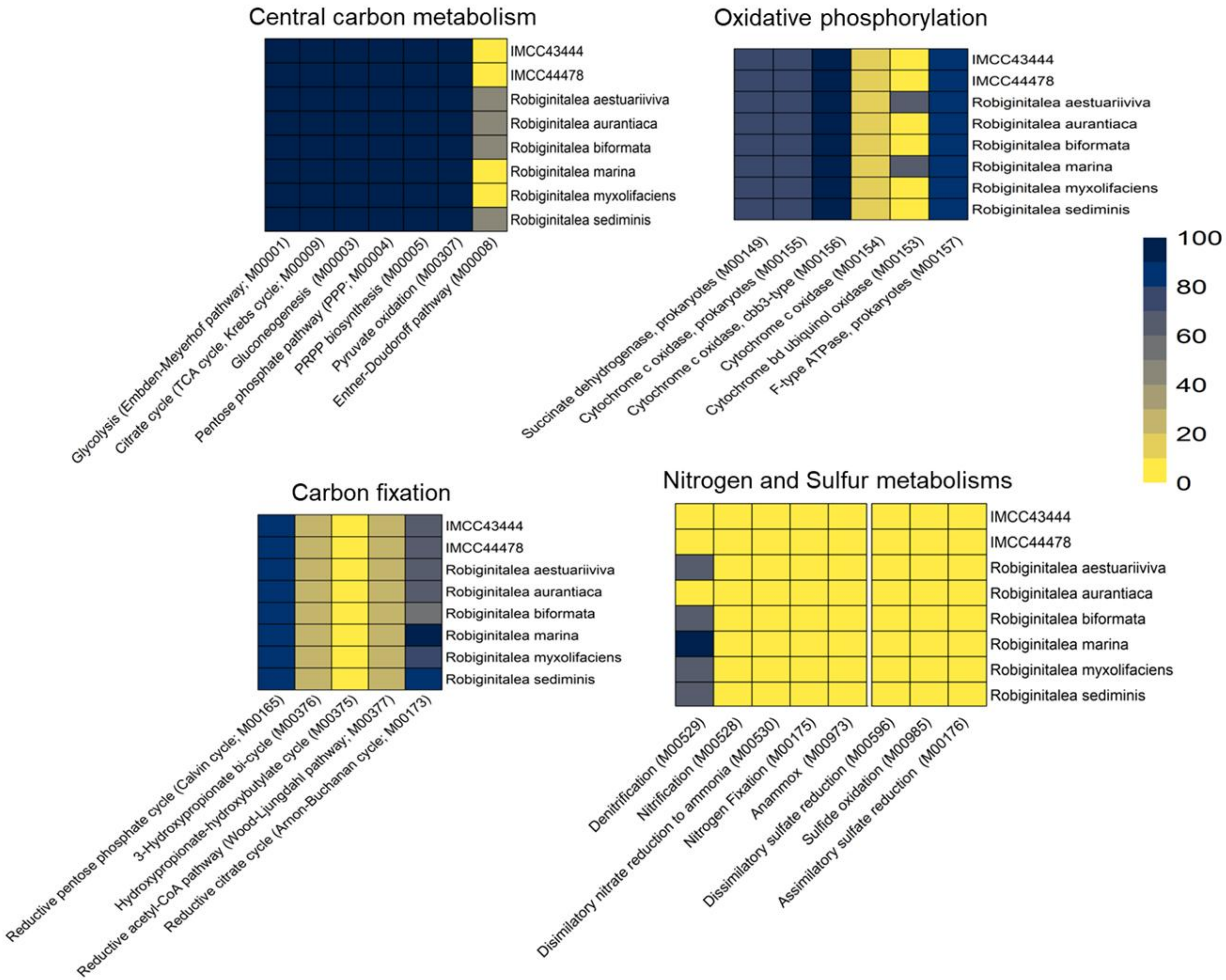

Supplement: Supplementary file 1 [file jm-2512009-Supplementary-Fig-S1.pdf]
