# Supplementary material for: Robiginitalea rubriflava sp. nov. and Robiginitalea insularis sp. nov., isolated from coastal seawaters of the Yellow Sea
Source: J Microbiol. 2026 Jan 28;64(2):e2512009. doi: 10.71150/jm.2512009 (PMC13577070; doi:10.71150/jm.2512009)

Fig. S2. Transmission electron micrographs of cells of strains IMCC43444^{T} (A) and IMCC44478^{T} (B) after negative staining with 1.0% (w/v) uranyl acetate. Cells were grown on marine agar at 25°C for 5 days. Bar, 0.5 μm.

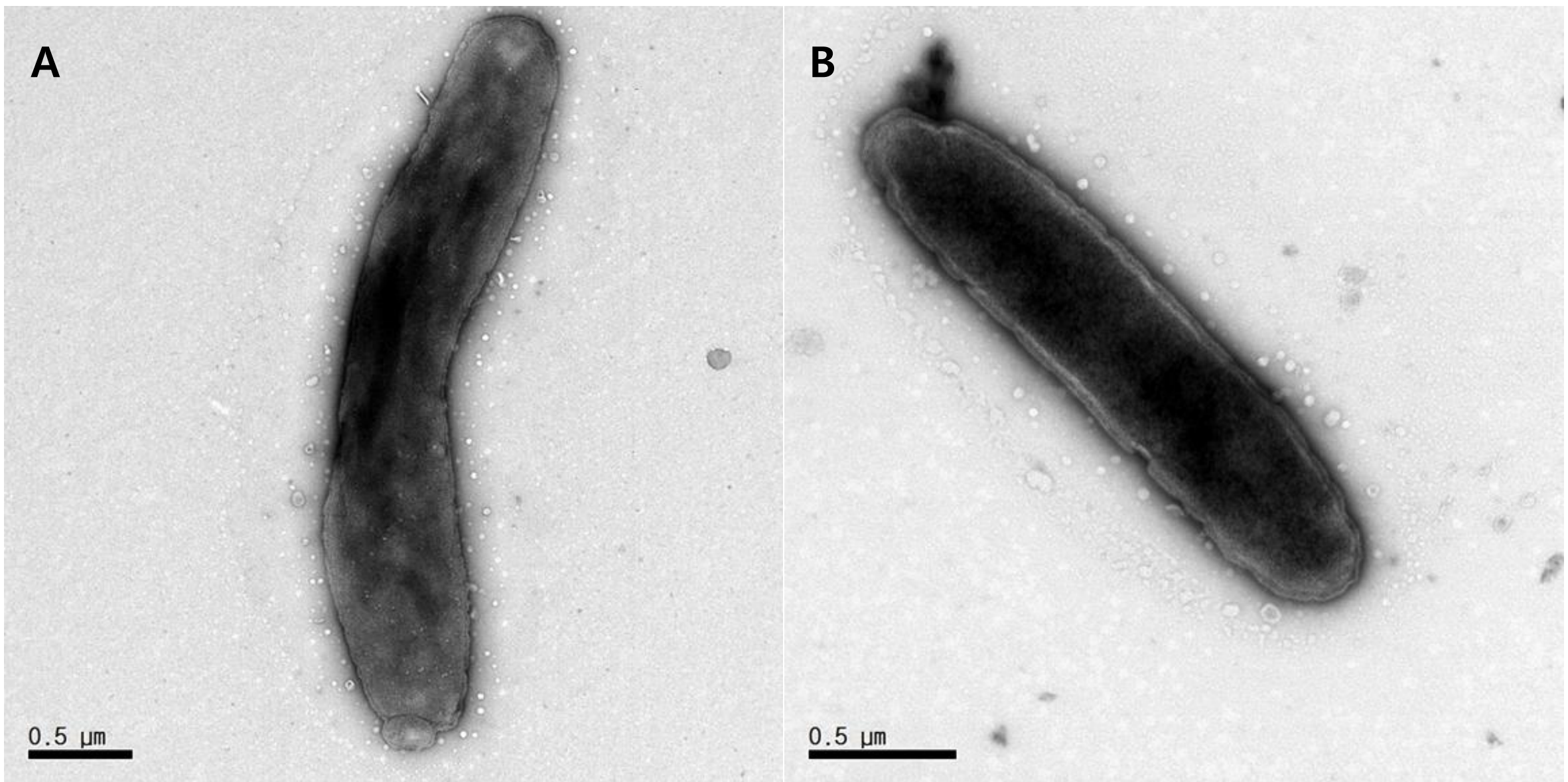

Supplement: Supplementary file 2 [file jm-2512009-Supplementary-Fig-S2.pdf]
