# Supplementary material for: Robiginitalea rubriflava sp. nov. and Robiginitalea insularis sp. nov., isolated from coastal seawaters of the Yellow Sea
Source: J Microbiol. 2026 Jan 28;64(2):e2512009. doi: 10.71150/jm.2512009 (PMC13577070; doi:10.71150/jm.2512009)

Fig. S3. Two-dimensional thin layer chromatograph of polar lipids of strains IMCC43444^{T} (A) and IMCC44478^{T} (B). PE, phosphatidylethanolamine; GL, unidentified glycolipid; AL, unidentified aminolipid; PL, unidentified phospholipid; L, unidentified lipid.

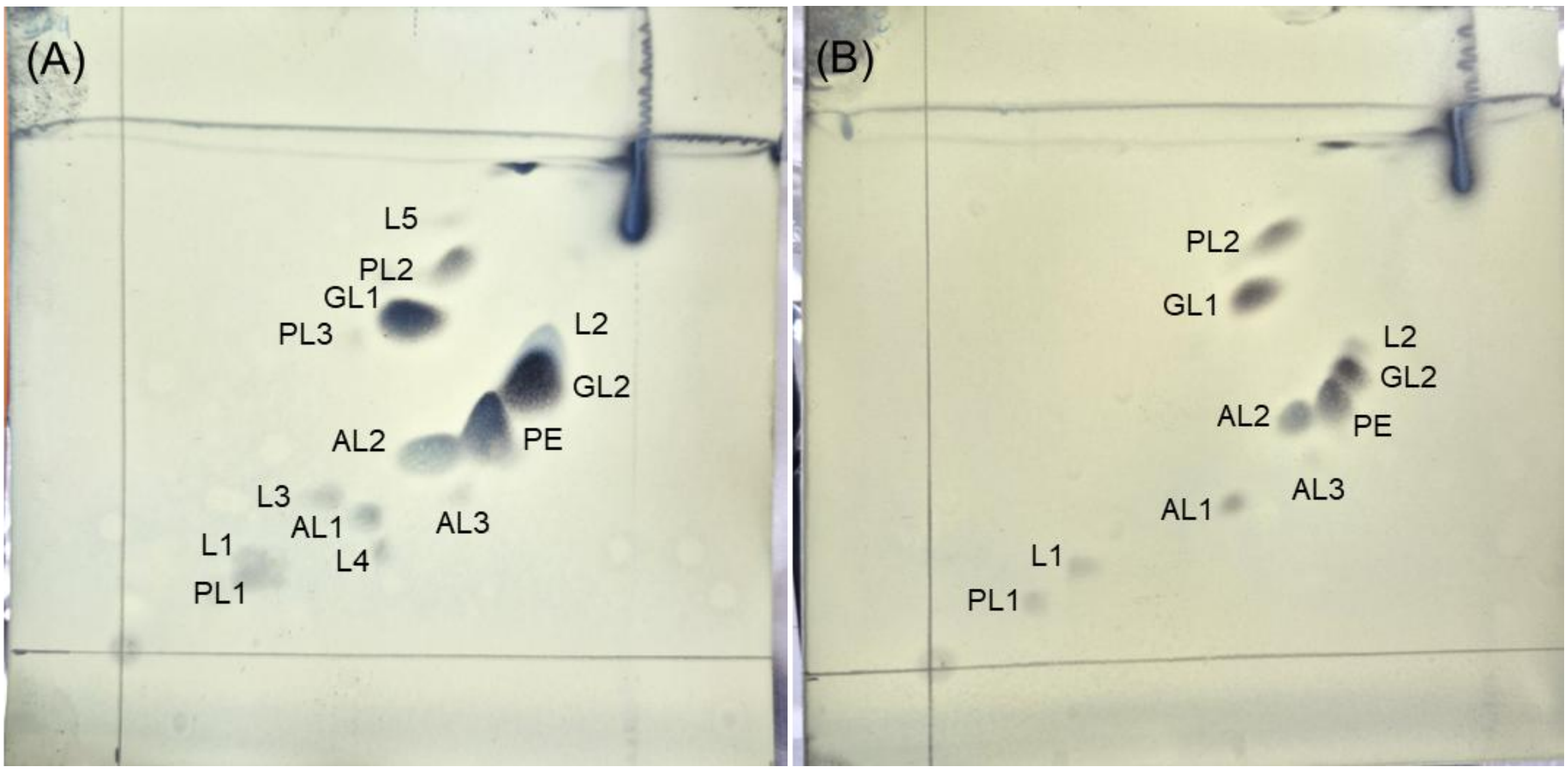

Supplement: Supplementary file 3 [file jm-2512009-Supplementary-Fig-S3.pdf]
