# Supplementary material for: Robiginitalea rubriflava sp. nov. and Robiginitalea insularis sp. nov., isolated from coastal seawaters of the Yellow Sea
Source: J Microbiol. 2026 Jan 28;64(2):e2512009. doi: 10.71150/jm.2512009 (PMC13577070; doi:10.71150/jm.2512009)
Supplement: Supplementary file 4 [file jm-2512009-Supplementary-Table-S1.pdf]

Table S1. Comparative genomic features of IMCC43444^T, IMCC44478^T and six validly published *Robiginitalea* species

Strains: 1, IMCC43444^T; 2, IMCC44478^T; 3, *R. biformata* HTCC2501^T; 4, *R. myxolifaciens* DSM 21019^T; 5, *R. sediminis* O458^T; 6, *R. marina* 2V75^T; 7, *R. aurantiaca* M39^T; 8, *R. aestuariiviva* M366^T.

Strain	1	2	3	4	5	6	7	8
GenBank accession number	CP199309	CP199296	CP001712*	FOYQ00000000*	NGNR00000000*	JAMXIB000000000*	JAUDUY000000000*	JARPUQ000000000*
Size (bp)	3,209,214	3,300,668	3,530,383	3,206,962	3,302,432	3,249,261	3,462,382	3,225,479
Number of contigs	1	1	1	3	15	49	52	4
G + C content (%)	46.5	46.4	55.3	48.9	55	54.8	46.9	55.7
Number of tRNA	39	40	41	37	39	39	38	38
Number of rRNA	6	6	6	3	3	3	3	3
Protein coding sequence	2,887	2,968	3,116	2,834	2,941	2,872	3,035	2,848

*Genomes of *R. biformata* HTCC2501^T, *R. myxolifaciens* DSM 21019^T, *R. sediminis* O458^T, *R. marina* 2V75^T, *R. aurantiaca* M39^T, and *R. aestuariiviva* M366^T were downloaded from NCBI GenBank database and annotated in this study for comparative genomic analysis.
