# Supplementary material for: Robiginitalea rubriflava sp. nov. and Robiginitalea insularis sp. nov., isolated from coastal seawaters of the Yellow Sea
Source: J Microbiol. 2026 Jan 28;64(2):e2512009. doi: 10.71150/jm.2512009 (PMC13577070; doi:10.71150/jm.2512009)
Supplement: Supplementary file 5 [file jm-2512009-Supplementary-Table-S2.pdf]

Table S2. Carbon source oxidation patterns (GENIII MicroPlate) of IMCC43444^T, IMCC44478^T, *Robiginitalea biformata* KCTC 12146^T, and *Robiginitalea sediminis* KCTC 52898^T

Strains: 1, IMCC43444^T; 2, IMCC44478^T; 3, *R. biformata* KCTC 12146^T; 4, *R. sediminis* KCTC 52898^T.

All data were obtained in this study. +, positive; –, negative.

Oxidation of carbons	1	2	3	4
Dextrin	–	+	+	+
D-Maltose	–	–	–	+
D-Trehalose	+	+	–	+
D-Cellobiose	–	–	–	+
Gentiobiose	+	+	+	+
Sucrose	–	–	–	+
D-Turanose	+	+	+	+
Stachyose	–	–	–	–
D-Raffinose	–	–	–	–
α-D-Lactose	–	–	–	–
D-Melibiose	–	–	–	–
β-Methyl-D-glucoside	–	–	–	+
D-Salicin	–	–	–	–
N-Acetyl-D-glucosamine	–	–	–	–
N-Acetyl-β-D-mannosamine	–	–	–	–
N-Acetyl-D-galactosamine	–	–	–	–
N-Acetyl neuraminic acid	–	–	–	–
α-D-Glucose	+	+	+	+
D-Mannose	–	–	–	–
D-Fructose	–	–	–	–
D-Galactose	–	–	–	–
3-Methyl-D-glucose	–	–	–	–
D-Fucose	–	–	–	–
L-Fucose	–	–	–	–
L-Rhamnose	–	–	–	–
Inosine	–	–	–	–
D-Sorbitol	–	–	–	–
D-Mannitol	–	–	–	–
D-Arabitol	–	–	–	–
myo-Inositol	–	–	–	–
Glycerol	–	–	–	–

D-Glucose-6-phosphate	–	–	–	–
D-Fructose-6-phosphate	–	+	+	+
D-Aspartic acid	–	–	–	–
D-Serine	–	–	–	–
Gelatin	–	–	–	–
Glycyl-L-proline	–	–	–	–
L-Alanine	–	–	–	–
L-Arginine	–	–	–	–
L-Aspartic acid	–	–	–	–
L-Glutamic acid	–	–	–	–
L-Histidine	–	–	+	–
L-Pyroglutamic acid	–	–	–	–
L-Serine	–	–	–	–
Pectin	–	–	–	–
D-Galacturonic acid	–	–	–	–
L-Galactonic acid lactone	–	–	+	–
D-Gluconic acid	–	–	–	–
D-Glucuronic acid	–	–	+	+
Glucuronamide	+	+	+	+
Mucic acid	–	–	–	–
Quinic acid	–	–	–	–
D-Saccharic acid	–	–	–	–
p-Hydroxyphenylacetic acid	–	–	–	–
Methyl pyruvate	–	–	–	–
D-Lactic acid methyl ester	–	–	–	–
L-Lactic acid	–	–	–	–
Citric acid	–	–	–	–
α-Ketoglutaric acid	+	–	+	+
D-Malic acid	–	–	–	–
L-Malic acid	+	+	+	+
Bromo-succinic acid	+	+	+	+
Tween 40	–	–	–	–
γ-Aminobutyric acid	–	–	–	–
α-Hydroxybutyric acid	–	–	–	–
β-Hydroxy-D,L-butyric acid	–	–	–	–
α-Ketobutyric acid	–	–	–	–
Acetoacetic acid	+	+	+	+
Propionic acid	–	–	–	–
Acetic acid	–	–	–	–
Formic acid	–	–	–	–
